# Development and Evaluation of a Reconstitutable Dry Suspension to Improve the Dissolution and Oral Absorption of Poorly Water-Soluble Celecoxib

**DOI:** 10.3390/pharmaceutics10030140

**Published:** 2018-08-29

**Authors:** Hye-In Kim, Sang Yeob Park, Seok Ju Park, Jewon Lee, Kwan Hyung Cho, Jun-Pil Jee, Hee-Cheol Kim, Han-Joo Maeng, Dong-Jin Jang

**Affiliations:** 1 Department of Pharmaceutical Engineering, Inje University, Gimhae 50834, Korea; hyein@bcwp.co.kr; 2 Institute of Digital Anti-Aging Healthcare, Inje University, Gimhae 50834, Korea; heeki@inje.ac.kr; 3 Samyang Biopharmaceuticals Corporation, Seongnam 13488, Korea; sangyeob.park@samyang.com; 4 Division of Nephrology, School of Medicine, Inje University, Busan 47392, Korea; medipark@inje.ac.kr; 5 Department of Nano Science and Engineering, Gimhae 50834, Korea; jwlee@inje.ac.kr; 6 Department of Pharmacy, College of Pharmacy, Inje University, Gimhae 50834, Korea; chokh@inje.ac.kr; 7 Department of Pharmacy, College of Pharmacy, Chosun University, Gwangju 61452, Korea; jee@chosun.ac.kr; 8 College of Pharmacy, Gachon University, Incheon 21936, Korea

**Keywords:** reconstitutable, nanosuspension, bead milling, crystallinity, dissolution, pharmacokinetics

## Abstract

This study aims at developing and evaluating reconstitutable dry suspension (RDS) improved for dissolution rate, oral absorption, and convenience of use of poorly water-soluble celecoxib (CXB). Micro-sized CXB particle was used to manufacture nanosuspension by using bead milling and then RDS was made by spray-drying the nanosuspension with effective resuspension agent, dextrin. The redispersibility, morphology, particle size, crystallinity, stability, dissolution, and pharmacokinetic profile of the RDS were evaluated. RDS was effectively reconstituted into nanoparticles in 775.8 ± 11.6 nm. It was confirmed that CXB particles are reduced into needle-shape ones in size after the bead-milling process, and the description of CXB was the same in the reconstituted suspension. Through the CXB crystallinity study using differential scanning calorimetry (DSC) and XRD analysis, it was identified that CXB has the CXB active pharmaceutical ingredient (API)’s original crystallinity after the bead milling and spray-drying process. In vitro dissolution of RDS was higher than that of CXB powder (93% versus 28% dissolution at 30 min). Furthermore, RDS formulation resulted in 5.7 and 6.3-fold higher area under the curve (AUC_∞_) and peak concentration (*C*_max_) of CXB compared to after oral administration of CXB powder in rats. Collectively, our results suggest that the RDS may be a potential oral dosage formulation for CXB to improve its bioavailability and patient compliance.

## 1. Introduction

Celecoxib (CXB) is a cyclooxygenase-2 selective and non-steroidal anti-inflammatory drug for osteoarthritis and rheumatoid arthritis [1,2]. It is known that CXB, a type II drug in biopharmaceutical classification system (BCS), is very poor in dissolution and oral absorption due to low solubility (3.2 μg/mL in water), in spite of its own therapeutic effect [3,4]. A version of the market product for CXB, Celebrex^®^(Pfizer) was designed to improve solubility and oral bioavailability through use of micronized CXB powder and solubilizer [5,6]. However, sodium lauryl sulfate (SLS) used as a solubilizer in Celebrex^®^, is known as toxic material, causing skin irritation and mucosal irritation due to it being an anionic surfactant with a high solubilizing potential [7,8]. Therefore, it is necessary to develop the formulation for improving oral absorption without using a very toxic ionic surfactant [9,10]. In addition, celecoxib used in high-dose administration (i.e., 200–400 mg twice daily) requires improvement for oral absorption and biocompatibility through novel dosage form, and it would help to improve greatly patient compliance [11]. To overcome these issues of CXB, various strategies such as self-emulsifying drug delivery system [3], solid dispersion [4], lipid carrier [12], and dry elixir [13] have been reported. The solubilizing assistants such as oil, hydrophilic polymer, lipid, and ethanol were used in micro-emulsion [3], matrix [4], lipid particle [12], and elixir system [13], respectively. If there are ways to expect the effective solubilization of poorly water-soluble CXB without the use of additional substances, it might be one of the useful approaches.

Recently, nanotechnology has been used as an effective strategy for drug delivery of poorly water-soluble materials [14,15,16]. When drug particles are made in nano-scale, their surface area is dramatically increased, resulting in great improvement in solubility, dissolution rate, and bioavailability, and there have been a variety of methods to do so [17,18,19]. Wet media milling (WMM) performed by physical force (e.g., shearing, impact, crushing, or attrition) is an efficient process to reduce the size of drug particles in aqueous media [20]. Nanosuspension manufactured by WMM, is available to greatly decrease the amount of additive used in manufacturing, and can be used for products with high drug amounts and low side effects, and is convenient for large-scale production [17,21]. However, in its long-term storage, drug particles grow or aggregate, causing frequent irreversible sedimentation [22]. This physical unstability has been mentioned as a big problem in developing a commercial product by applying nanosuspension technology [23,24]. Even though studies to develop nanosuspension for the poorly water-soluble drug have been invigorated [25,26], there have been very few research results to improve unstability of nanosuspension [27]. According to some literature, nanosuspension was made in solid dosage form through spray-drying, freezing drying, pelletization, and granulation to improve stability and the drug was administrated to patients by reconstituting it from aqueous solution [28,29,30]. However, processes used for manufacturing the solid dosage form add various stresses on the drug, and as a result, may accompany irreversible aggregation of nanoparticles, requiring investigation [22,23].

The purpose of this study was to develop and evaluate a reconstitutable dry suspension (RDS), without anionic surfactant used in the commercial product, in order to improve the dissolution and oral absorption of celecoxib. To the best of our knowledge, no scientific literature is currently available on the improvement of in vitro dissolution profiles and in vivo pharmacokinetics of CXB by RDS formulation. We respectively utilized bead-milling equipment and a spray dryer for the size reduction and solidification process, and used dextrin as a solid matrix material. Dextrin has various advantages as a pharmaceutical excipient. Dextrin is known to be eligible as an immediate-dissolving solid dosage formulation forming polymer due to its non-toxic, high water-soluble, and biocompatible properties [31,32]. Dextrin is an excellent wall-forming material for micro-encapsulation of core materials [33,34]. Unlike many other polymers, solid dosage forms made of dextrin have non-sticky and free-flowing properties [31,32], which can be beneficial to further unit operations after the drying process. The redispersibility, morphology, crystallinity, physical stability, dissolution, and pharmacokinetic profile of celecoxib nanosuspension or RDS were characterized.

## 2. Materials and Methods

### 2.1. Materials

CXB (Crystal Form III) was purchased from MYLAN (Andhra Pradesh, India). Dextrin and Tween 80 were purchased by Daejung (Siheung, Korea) and Duksan (Ansan, Korea), respectively. All other chemicals and solvents were of analytical reagent grade.

### 2.2. Preparation of Nanosuspension and Dry Suspension

#### 2.2.1. Preparation of Nanosuspension Using Wet Bead Milling

Tween 80 as a stabilizer was dissolved in de-ionized water (0.5% *w*/*v*). CXB was carefully added to the aqueous solution (2% *w*/*v*) and dispersed using MTOPS MS-3040 mechanical mixer (Seoul, Korea). The resulting suspension was loaded into the chamber of a Netzsch bead mill (Minicer, Germany) for size reduction process. In order to prevent screen clogging, the flow was gradually increased from 10 mL/min. The operating conditions for bead milling are as follows: circulation flow, 100 mL/min; milling speed, 3000 rpm; milling time, 4 h; and product temperature, 17–20 °C. The milling machine equipped with Yttrium-stabilized zirconia beads (0.3 mm diameter) as a milling media was operated in a recirculation mode.

#### 2.2.2. Preparation of Dry Suspension Using Spray-Drying

The homogenized nanosuspension (containing an amount equivalent to 1 g CXB) was blended with dextrin (20 g) as the matrix material. The resulting mixture was spray-dried using Büchi B290 mini Spray Dryer (Flawil, Switzerland) under the following parameters: Inlet air temperature, 120 °C; outlet air temperature, 68–70 °C; spray flow control, 470 NL/h; sample feeding flow, 3 mL/min; and aspiration, 100% [35]. The nanosuspension during spray-drying was continuously stirred with a magnetic stirrer. The spray-dried RDS powder was collected and kept in a sealed container at the refrigerator.

### 2.3. Characterizations of Nanosuspension and RDS

#### 2.3.1. Particle Size, Distribution and Zeta Potential Analysis

The particle size and distribution of drug nanoparticles in the formulation was determined by Brookhaven 90 Plus dynamic light scattering particle size analyzer (Holtsville, NY, USA). All data were recorded as volume distributions under a scattering angle of 90° at 25 °C. The sample was diluted with de-ionized water and vortexed (30 s) before measurement. The width of the particle size distribution was calculated using SPAN value and polydispersity index (PDI). *D*_0.1_, *D*_0.5_, and *D*_0.9_ are the size at 10%, 50%, and 90% of the cumulative volume, respectively. The zeta potential was measured using a Brookhaven NanoBrook 90 Plus zeta potential analyzer (Brookhaven Instruments Corp., Holtsville, NY, USA).

SPAN = [*D*_0.9_ − *D*_0.1_]/*D*_0.5_ (1)

#### 2.3.2. Redispersibility

The redispersibility of the RDS was evaluated using redispersibility index (RDI). In a glass vial, the RDS was mixed with distilled water and vortexed (30 s). RDI is defined as the following equation. *D* and *D*_0_ are the mean particle size of reconstituted suspension and nanosuspension before spray-drying, respectively [36]. When the RDI value is near to 100%, it means that the dried suspension powder can be completely redispersed to nanoparticle before spray-drying.

RDI = [*D*/*D*_0_] × 100% (2)

#### 2.3.3. Scanning Electron Microscopy (SEM)

The morphologies of CXB powder, nanosuspension, RDS, and reconstituted suspension were observed using Hitachi S-4300SE FE-SEM (Tokyo, Japan). The samples were put on a double-faced carbon tape and air dried at 30 °C. The resulting samples were coated with platinum and examined at an accelerating voltage of 15 kV.

#### 2.3.4. Solid-State Characterization

The solid-state characterizations of nanosuspension and RDS were conducted by differential scanning calorimetry (DSC) and powder X-ray diffractometry (PXRD). The thermal transition patterns of various samples were obtained using a TA Q20 differential scanning calorimeter (Leatherhead, UK). The samples (approximately 3 mg) were weighed in an aluminum pan and sealed with a lid. The pretreated cans were scanned from 30 °C to 200 °C at a rate of 10 °C/min under a continuous flow of dried nitrogen gas. X-ray diffraction patterns were gained using Rigaku Ultima IV X-ray diffractometer (Akishima, Japan) with Cu-Kɑ radiation. The samples were gently mounted on a sample holder and PXRD patterns were collected over a range of 3° to 40° (2θ) using scanning speed of 2.0° per min and a step size of 0.02° [37].

#### 2.3.5. Physical Stability

The physical stability of the nanosuspension and RDS were determined at the predetermined time intervals after being stored in a sealed vial protected from the outer atmosphere at room temperature. The appearance observation, particle size, distribution, redispersibility, and zeta potential of samples were monitored for 12 weeks.

#### 2.3.6. In Vitro Dissolution Test

Dissolution of CXB powder, marketed product, nanosuspension, and RDS were performed using Electrolab TDT-08 L Dissolution Tester (Mumbai, India). The dissolution study was conducted at 36.5 ± 0.5 °C with a paddle speed of 50 rpm. Each sample containing an amount equivalent to 2.5 mg CXB was uniformly dispersed in 900 mL dissolution media (containing 0.1% Tween 80) of pH 1.2 (gastric fluid) and pH 6.8 (intestinal fluid). Three milliliters of each medium were collected at 5, 10, 15, 30, 60, 90, 120 min and replaced by an equivalent volume of fresh dissolution medium. The obtained samples were filtered using a 0.45 µm membrane filter and analyzed at 250 nm. The CXB content was quantified using an Agilent 1100 HPLC system (Santa Clara, CA, USA) equipped with a UV detector and Young Jin Biochrom Aegispak C18 column (4.6 × 150 mm, 5 µm, Seongnam, Korea). The mobile phase consisted of MeOH and H_2_O (75:25, *v*/*v*) and used at a flow rate of 1.25 mL/min. Dissolution was performed in triplicate [38].

#### 2.3.7. In Vivo Oral Pharmacokinetic Studies in Rats

The in vivo oral pharmacokinetic studies of CXB powder, RDS, and Celebrex^®^ (commercial product) were investigated at a dose of 5 mg/kg in the fasted condition of male Sprague Dawley (SD) rats. All animal experiments were performed in accordance with the Guidelines for Animal Care and Use issued by Gachon University, as described previously [39]. Experimental protocols involving the animals used in this study were reviewed and approved by the Animal Care and Use Committee of the Gachon University (#GIACUC-R2018004, approval date on 11 May 2018). The animals were fasted overnight (i.e., 18 h before oral administration) but allowed to water. After rats were anesthetized with Zoletil (20 mg/kg, intramuscular injection), femoral arteries were cannulated for blood sample collection with a Clay Adams PE-50 polyethylene tube (Parsippany, NJ, USA) filled with heparinized saline (20 IU/mL). After recovery from surgery, rats were orally administered at a dose of 5 mg/kg of celecoxib all for tested three formulations. Blood samples (100 µL) were collected at 0, 15, 30, 60, 120, 180, 240, 360, 480, 1440, and 2880 min after administration. Plasma was obtained by centrifuging whole blood at 4 °C for 10 min and then stored at −20 °C.

Sample preparation was performed by deproteinizing with 100 µL of methanol solution containing internal standard (IS). Upon vortex-mixing, centrifugation at 12,000× *g* at 4 °C for 10 min was performed and then the supernatant was obtained. A liquid chromatography tandem mass spectrometry (LC–MS/MS) bioanalytical method was applied with a simple modification from a previous method reported [40]. In detail, the LC-MS/MS system consisted of an Agilent HPLC and Agilent 6460 QQQ mass spectrometer with ESI^+^ Agilent Jet Stream ion source (Santa Clara, CA, USA). The separation of celecoxib and IS was achieved from endogenous plasma substances with Phenomenex Synergi 4μ polar-RP 80A column (150 × 2.0 mm, 4 µm, Torrance, CA, USA) using the mixture of 0.1% formic acid and methanol (65:35, *v*/*v*) at 0.2 mL/min of flow rate. For the quantification of CXB and IS, multiple reaction monitoring (MRM) in the positive electrospray ionization (ESI^+^) mode were chosen, for which the parent ion to production ion transitions were as follows: celecoxib, 381.9→362.0; IS (atorvastatin), 559.2→440.2.

Based on observed plasma concentration-time profiles, the peak concentration (*C*_max_) and time to reach *C*_max_ (*T*_max_) were read directly. Other pharmacokinetic parameters were calculated by non-compartmental analysis using Pharsight Winnonlin 5.0.1 (Cary, NC, USA), as described previously [39]. Furthermore, the relative oral bioavailability (BA) was calculated by dividing AUC after oral administration of dried nanosuspension or commercial product by AUC after oral administration of CXB powder. Statistically significant differences were indicated by *p*-value of <0.05 based on a *t* test between two means for unpaired data or a Duncan’s multiple range test posteriori analysis of variance (ANOVA) among three means for unpaired data.

## 3. Results and Discussion

### 3.1. Morphology of Nanosuspension and Reconstitutable Dry Suspension 

**Figure 1:**
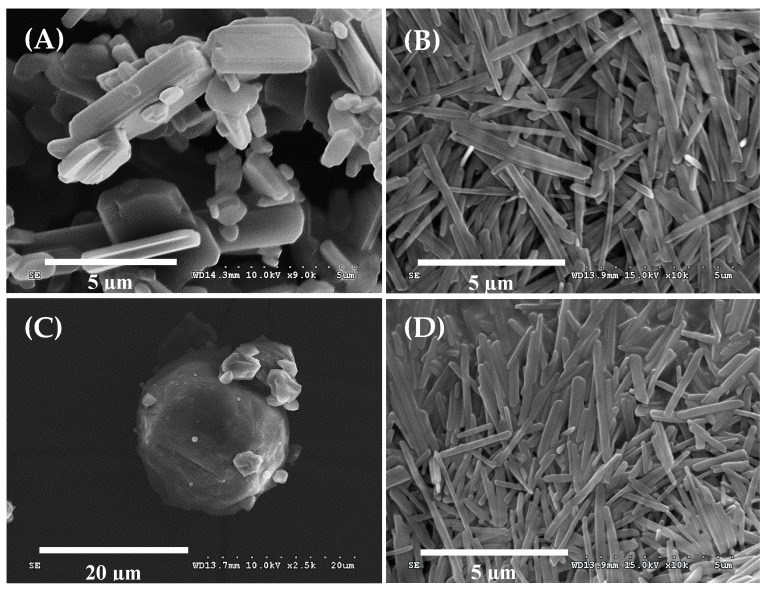
SEM images of (**A**) celecoxib (CXB) powder, (**B**) Nanosuspension, (**C**) reconstitutable dry suspension (RDS), and (**D**) Reconstituted RDS in aqueous solution.

In this study, RDS was manufactured by reducing CXB particles in size through bead milling and spray-drying. The encapsulated CXB content (4.2 ± 0.2%) appeared to be uniformly maintained in RDS. The particle morphology of the CXB powder, nanosuspension, and RDS by SEM is shown in Figure 1. CXB particles (Figure 1A, mean particle size, 6.4 μm) were changed to rod-shaped ones after the milling process (Figure 1B). The milled particles in this study, unlike the previous studies with mainly spherical or plate-like ones, showed different shapes [9,41]. It is known that the description of the particles is closely related to diameters of API and bead used in the milling process, but its precise correlation is not revealed, yet [42]. In this study, the particles were rod-shaped, and as a result, depending on the direction of observation, their lengths seemed very different (Figure 1B). The short axis of the milled particles was very small, around 200–300 nm while the long axis was comparatively large, about 2–3 μm.

The nanosuspension manufactured through the milling process was smoothly converted into RDS through spray-drying. The outer morphology of the RDS was spherical with a smooth surface, and small pieces of particles clung to large particles (Figure 1C). It was also observed that the spherical microparticles were relatively free-flowing. In this study, to manufacture RDS, dextrin was selected as an effective diluent to compose a matrix. It has been used as a polymeric carrier in various kinds of solid dosage forms, since it is stable and compatible with hydrophilic or hydrophobic material and also has good free-flowing and water-soluble properties suitable for improved dissolution or reconstitution of the solid dosage form [31,32]. Due to these merits of dextrin, it was confirmed that RDS could be perfectly recovered to nanosuspension before the spray-drying process as it was well reconstituted in water (Figure 1D).

In the fabrication using the wet milling process, an appropriate stabilizer must be used to disperse and stabilize the nanosuspension. Hydrophilic polymers or non-ionic surfactants such as Pluronic^®^, Span, Tween, TPGS, HPC, and HPMC have been used as stabilizers in many cases [43]. More recently, advanced research based on surfactant-free nanosuspension has been reported using superdisintegrants instead of conventional stabilizers [10,44,45]. According to our preliminary study (data not shown), when using a hydrophilic stabilizer, HPMC, the formation and dispersion of nanosuspension were found to be good, but the reconstitution behaviors of RDS after the drying process were observed to be poor. After confirming these results, various additives were also tested in terms of the milling process and redistribution step. As a result, it was confirmed that Tween 80 can be effectively used as a stabilizer in very small amounts.

### 3.2. Characterizations of Suspension System

#### 3.2.1. Particle Size and Redispersibility

**Figure 2:**
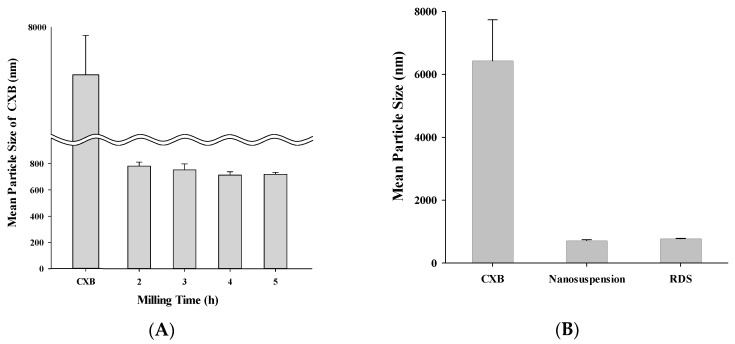
Mean particle size of (**A**) nanosuspension during milling process; (**B**) CXB powder, nanosuspension, and reconstituted RDS by photon correlation spectroscopy.

It is important to set optimal process time in milling for size reduction of solid particles [46,47]. If the milling time is too short, milling is not properly done, resulting in difficulty getting small and homogeneous particles, while a milling time that is too long may cause changes in internal structures, such as crystallinity of particle [25]. Accordingly, in this study, by monitoring size and crystallinity during the milling process, optimal process time was determined (Figure 2A). CXB powder (6.4 ± 1.3 µm) is decreased in size according to milling time and it was decreased 781.4 ± 31.2 nm after two hours and 713.0 ± 25.3 nm after four hours, respectively. However, after some time, size was not decreased anymore. Therefore, for manufacturing small dispersion system, it was judged that 4 h milling time was the best condition. Each SPAN and PDI value of nanosuspension manufactured through 4 h milling was 1.31 and 0.24, showing comparatively narrow size distribution and zeta potential was −19.0 ± 1.9 mV, showing its appropriateness as suspension (Table 1). It is known that a suspension keeps its stable state when its zeta potential is over 10 mV in absolute value [48].

After reconstitution, checking particle size, SPAN, PDI, RDI, and zeta potential of the nanosuspension, redispersibility of RDS was evaluated. CXB of around 6400 nm was decreased to 713.0 ± 25.3 nm in the form of nanosuspension, and after dried as RDS and reconstituted, the size was not different, at 775.8 ± 11.6 nm (Figure 2B). The SPAN and PDI values of the reconstituted nanosuspension were 1.32 and 0.26, and the RDI calculated through changes of particle size was 108, showing similar value compared to nanosuspension before its drying (Table 1). A high SPAN or PDI value represents a wide distribution in size, whereas a low value represents a narrow distribution [49,50]. The zeta potential of RDS was −15.8 ± 1.5 mV, which was also similar to the initial value (−19.0 ± 1.9 mV). These results indicate that the RDS entrapping CXB nanosuspension could be dispersed well on reconstitution with aqueous solution, resulting nanosuspension with a slightly larger mean particle size than the milled nanosuspension before spray-drying process. The slight increase in the mean particle of dispersed system size may be elucidated by the formation of larger particles due to coalescence or aggregation during the spray-drying process as reported in the previous study [35]. Our data show that the RDS can be efficiently reformed into nanosuspension upon reconstitution without any change of characteristics such as particle size, distribution, RDI, and zeta potential.

#### 3.2.2. Solid-State Characterization

Internal structures of the solid could be changed by high energy and heat during the milling and spray-drying processes, causing problems in quality of pharmaceutical products [51]. Microscopy, DSC, and PXRD technique are frequently applied for internal structure determination of pharmaceutical solids [52,53,54,55]. We also thought that the influences of fabrication processes and excipients might lead to changes in melting or crystal form of CXB within the dried formulation. Therefore, maintenance of internal structure of CXB, the crystallinity in RDS was investigated by using SEM, DSC, and PXRD. As shown in Figure 1, the physical appearances of reconstituted nanosuspension and the nanosuspension before drying were found to be identical (via SEM images).

**Figure 3:**
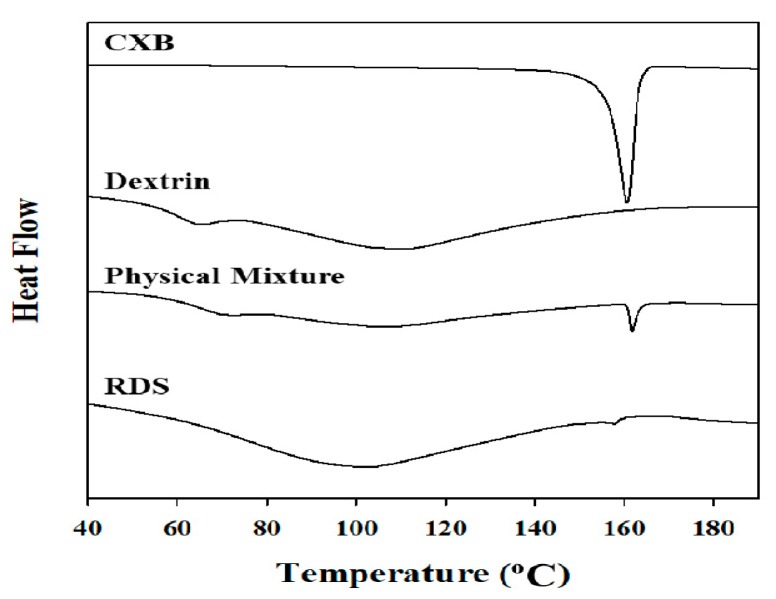
Differential scanning calorimetry (DSC) thermograms of CXB powder, dextrin, physical mixture, and RDS.

**Figure 4:**
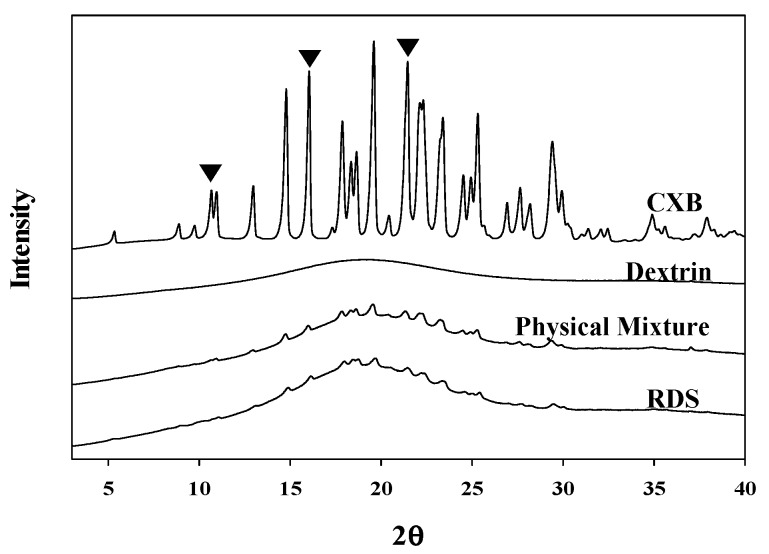
X-ray diffractograms of CXB powder, dextrin, physical mixture, and RDS.

The DSC thermograms (Figure 3) showed that the melting (endothermic) peak at 159–161 °C of CXB observed in the physical mixture and RDS formulation (CXB: Dextrin = 1:20, *w*/*w*). However, the peak in RDS was very small and broad, compared to that in physical mixture. Unlike physical mixture (with dextrin and CXB), Tween 80 as a stabilizer was additionally added in RDS, and it is presumed that peak size was smaller due to the influence of the stabilizer used [56]. The several previous investigations have also reported about the results of smaller melting peak of encapsulated drug due to the influence of Tween 80 as surfactant included in solid dosage form. Regardless of solid-state of drug in the solid matrix, this phenomenon is likely due to the effect of Tween 80 when the crystal lattice of drug absorbs heat and dissolves [57,58]. CXB has a drug with polymorphism, and in this study, form III was used. It is known that CXB form III has clear peak in specific 2θ (10.5°, 16°, 21.5°) [51,59]. In the X-ray diffractograms shown in Figure 4, crystalline peaks of CXB were clearly observed in the physical mixture and RDS. Unlike DSC analysis, the specific peaks representing CXB in X-ray diffractograms were almost identical in both physical mixture and RDS. Taken together, it is indicated that internal structure is not likely changed by milling, spray-drying, or Tween 80 used to manufacture nanosuspension and RDS, and the CXB’s crystallinity is consistently maintained.

#### 3.2.3. Physical Stability

**Figure 5:**
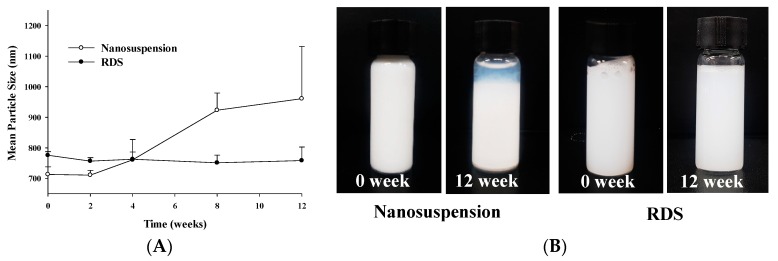
Stability data of nanosuspension and RDS for storage period: (**A**) Mean particle size and (**B**) photographs of physical appearance.

In spite of various advantages of nanosuspension, it is known that there are problems of physical unstability such as aggregation, sedimentation, and phase separation due to dramatic increases in surface and Brownian motion [10,60]. Therefore, nanosuspension should be tested for stability during its storage period. In this study, the respective stability of nanosuspension and RDS was checked during 12 weeks (Figure 5 and Table 1). It was confirmed that all particle size, SPAN, PDI, and zeta potential of nanosuspension were considerably changed. Particle size increased from 713.0 ± 25.3 nm to 965.9 ± 170.9 nm as time went by, while zeta potential decreased from −19.0 ± 1.9 mV to −7.7 ± 0.8 mV. The significant changes of several indices showing stability of nanosuspension were reflected in physical unstability such as sedimentation and phase separation of suspension system (Table 1 and Figure 5B). In contrast to nanosuspension with severe unstability, RDS showed improved stability in all the indices without notable changes. The particle sizes of RDS were 775.8 ± 11.6 nm (initial) and 758.7 ± 44.1 nm (12 weeks), and the zeta potentials were −15.8 ± 1.5 mV (initial) and −15.5 ± 2.5 mV (12 weeks), respectively, maintaining almost identical values. The RDI value is about 106−108%, which means that the redispersibility of the RDS is very good. These results obviously show that the RDS containing CXB can be reformed into initial nanosuspension upon reconstitution without any change of unstability index during storage.

### 3.3. Dissolution Study

**Figure 6:**
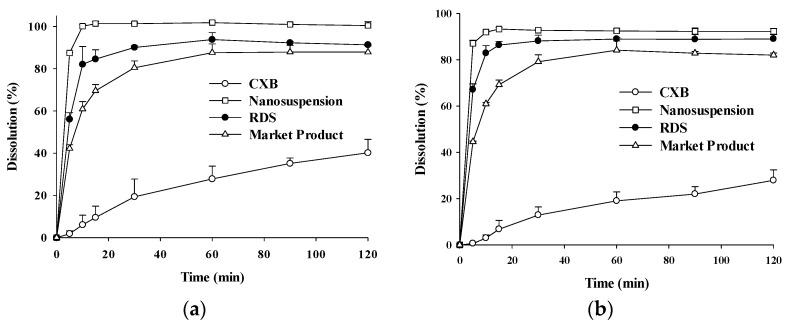
The dissolution profiles of CXB in (**A**) pH 1.2 and (**B**) pH 6.8 medium at 36.5 ± 0.5 °C from CXB, Nanosuspension, RDS, and marketed product.

The profiles of CXB dissolved from CXB powder, nanosuspension, RDS, and marketed product were shown in Figure 6. The dissolution profiles were found to be similar at pH 1.2 and pH 6.8. The aqueous solubility of CXB powder is very low about 3–7 μg/mL when analyzed in vitro [61]. Considering the pKa of CXB (i.e., 11.1), CXB solubility is not likely to vary from pH 1.2–8.0, corresponding to the in vivo range in the gastro-intestinal tract [61]. The amounts of CXB dissolved from reconstituted RDS were similar to those from nanosuspension, but higher than those of CXB powder and marketed product. In particular, dissolution rates of nanosuspension and RDS were very different from CXB powder. The dissolution profile of a marketed product (Celebrex^®^) also tended to be very high, compared to CXB powder. However, higher dissolution for nanosuspension is likely due to increased surface area through nano-milling process, whereas the dissolution of marketed product is high through operation of SLS included as ingredient in Celebrex^®^. It suggests that reconstituted of RDS without toxic SLS in aqueous solution efficiently formed a CXB nanosuspension with similar dissolution profiles to nanosuspension before spray-drying. The improved dissolution rates of CXB in water might be expected to contribute to the increase in oral absorption.

### 3.4. Pharmacokinetic Study

**Figure 7:**
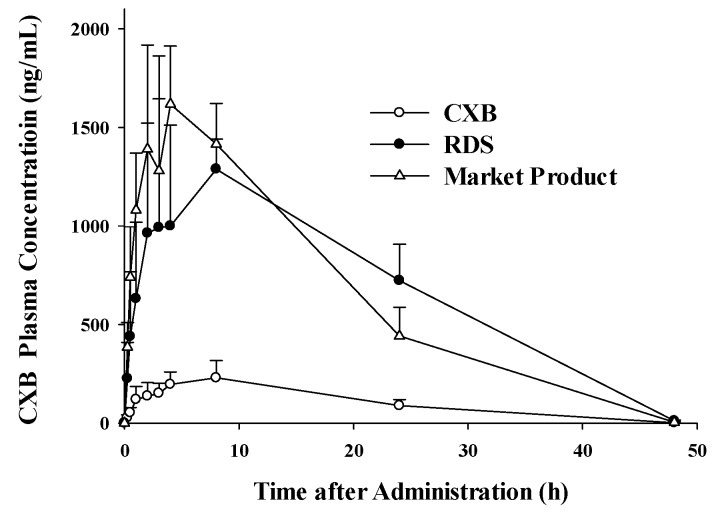
Plasma concentration profiles in the Sprague Dawley (SD) rats after oral administration of an equivalent dose of CXB (5 mg/kg), RDS, and marketed product (Celebrex^®^).

The pharmacokinetics of CXB powder, reconstituted RDS, and commercial product were investigated and compared in rats. Figure 7 shows temporal profiles of CXB concentrations in plasma after a single oral administration of the three formulations, respectively. The pharmacokinetic parameters, including *C*_max_, *T*_max_, *T*_1/2_, AUC_last_, AUC_∞_, and MRT of CXB are summarized in Table 2. Maximum concentration in plasma (*C*_max_) and the area under the curve (AUC_∞_) of the developed RDS were significantly increased by 5.7- and 6.3-fold, compared to the CXB powder group, which was comparable to the marketed product. Although our developed RDS formulation does not contain the anionic surfactant (i.e., SLS), unlike Celebrex^®^, it was comparable to the commercial product with respect to the systemic exposure (i.e., AUC) and *C*_max_, with similar relative BA (625% vs. 605%). Therefore, these pharmacokinetic observations strongly indicate that developed RDS exert dramatically enhanced oral absorption of CXB in vivo in rats. The needs for developing various approaches have been proposed to widen the application of CXB, a poorly water-soluble and high-dose drug (400 mg twice daily) [62]. Since commercial product (Celebrex^®^, Pfizer) is the capsule formulation containing SLS to increase the solubility and bioavailability of CXB, the alternative approach using reconstitutable dried nanosuspension, not using anionic SLS as a solubilizer, may be useful to widen the usage of CXB in terms of patient compliance and safety.

## 4. Conclusions

RDS formulation containing CXB was reconstituted well and stability indexes such as mean particle size, SPAN, PDI, RDI, appearance, and zeta potential were almost unchanged for storage period. The CXB encapsulated in RDS matrix retained its original crystallinity after milling and spray-drying processes. The RDS increased the dissolution of the encapsulated CXB and improved in vivo absorption in rats after oral administration compared unmilled powder. The in vivo absorption of CXB when administered in RDS was comparable to the marketed product, which contains anionic surfactant (SLS) to increase the solubility and absorption of CXB. Taken together, the RDS might be developed as an alternative delivery system to improve both the bioavailability and patient compliance.

## Figures and Tables

**Table 1:** Physical stability of nanosuspension and reconstitutable dry suspension (RDS) (*n* = 5, mean ± SD).

Formulation	Time (Week)	Particle Size (nm)	SPAN	PDI	RDI (%)	Zeta Potential (mV)
**Nano** **Suspension**	0	713.0 ± 25.3	1.31 ± 0.03	0.24 ± 0.01		−19.0 ± 1.9
	2	711.1 ± 15.5	1.26 ± 0.02	0.24 ± 0.01		
	4	760.9 ± 66.5	1.24 ± 0.03	0.23 ± 0.02		
	8	923.0 ± 56.4	1.14 ± 0.04	0.20 ± 0.03		
	12	965.9 ± 170.9	1.43 ± 0.06	0.35 ± 0.05		−7.7 ± 0.8
**RDS**	0	775.8 ± 11.6	1.32 ± 0.04	0.26 ± 0.04	108	−15.8 ± 1.5
	2	756.7 ± 11.0	1.38 ± 0.03	0.29 ± 0.02	106	
	4	762.9 ± 23.4	1.35 ± 0.03	0.28 ± 0.03	107	
	8	769.0 ± 5.00	1.40 ± 0.05	0.32 ± 0.03	108	
	12	758.7 ± 44.1	1.35 ± 0.03	0.25 ± 0.02	106	−15.5 ± 2.5

PDI: polydispersity index; RDI: redispersibility index; RDS: reconstitutable dry suspension.

**Table 2:** Pharmacokinetic parameters of celecoxib (CXB) following oral administration of CXB powder, RDS and a marketed product in rats (*n* = 4, mean ± SD).

Pharmacokinetic Parameters	CXB Powder	RDS	Market Product
*T*_max_ (min)	420 ± 69	405 ± 150	195 ± 57
*C*_max_ (μg /mL)	0.26 ± 0.08	1.49 ± 0.29	1.64 ± 0.31
*T*_1/2_ (min)	345 ± 70	357 ± 56	295 ± 66
AUC_last_ (μg∙min/mL)	304 ± 85	1904 ± 350	1844 ± 316
AUC_∞_ (μg∙min/mL)	305 ± 85	1910 ± 353	1847 ± 318
MRT (min) ^a^	789 ± 148	888 ± 22	684 ± 79
Relative BA (%)		625	605

^a^ MRT = AUMC/AUC; AUMC: area under the first moment curve; BA: oral bioavailability.
